# The effect of an extended culture period on birth weight among singletons born after single or double vitrified embryo transfer

**DOI:** 10.3389/fendo.2024.1184966

**Published:** 2024-03-19

**Authors:** Ningling Wang, Kaibo Lin, Xinxi Zhao, Ping Zhang

**Affiliations:** ^1^ Department of Assisted Reproduction, The International Peace Maternity and Child Health Hospital, School of Medicine, Shanghai Jiao Tong University, Shanghai, China; ^2^ Department of Assisted Reproduction, Shanghai Ninth People’s Hospital, School of Medicine, Shanghai Jiao Tong University, Shanghai, China

**Keywords:** birth weight, blastocyst transfer, neonatal outcome, vitrification, culture period

## Abstract

**Aim:**

To evaluate the effect of an extended culture period on birth weight among singletons born after vitrified-warmed embryo transfer

**Methods:**

A retrospective cohort study was performed among 12400 women who gave birth to 1015, 1027, 687, and 9671 singletons after single blastocyst transfer, single cleavage-stage embryo transfer, double blastocyst transfer, and double cleavage-stage embryo transfer, respectively.

**Results:**

The unadjusted birth weight of singletons born after vitrified blastocyst transfer were heavier than those born after cleavage-stage transfer (β=30.28, SE=13.17, P=0.022), as were the adjusted birth weights (β=0.09, SE=0.03, P=0.007). In addition, there was a 37% increased odd of having an infant with high birth weight after vitrified blastocyst transfer compared with vitrified cleavage stage transfer (OR=1.37, 95% CI:1.07-1.77).

**Conclusion:**

The unadjusted and adjusted birth weight and odds of having an infant with high birth weight significantly increased after blastocyst transfer compared with cleavage-stage embryo transfer in vitrified-warmed cycles.

## Introduction

Forty years after the birth of the first *in vitro* fertilization (IVF) baby in 1978, many new assisted reproductive technologies (ART) have been widely applied to clinical practice in the past decades. Along with the increasing development and maturity of cryopreservation techniques, frozen embryo transfer (FET) has been a routine procedure that increases the cumulative pregnancy rate and live birth rate per ovarian stimulation cycle and decreases the risk of ovarian hyperstimulation syndrome (OHSS) (1, 2). Blastocyst transfer has been popular worldwide, as it improves the implantation rate and live birth rate by providing better embryo-endometrium synchrony, more closely mimicking the sequence of events in natural conception, and increasing the chance of transferring a good-quality embryo (3). There is a global trend to reduce the number of transferred embryos aiming to minimize the multiple pregnancy rate and its associated complications (4). Single embryo transfer (SET) as a strategy has been advocated in clinically appropriate patients for years, and even mandated in many countries. Considering that the ultimate outcome of ART therapy should be a healthy singleton baby, not pregnancy or live birth, evaluating the safety of these important technologies regarding neonatal outcomes is necessary.

Birth weight is related to infants’ short and long-term health. Previous studies have reported that infants with low birth weight (LBW) have a higher risk of cardiovascular diseases in adulthood, and large-for-gestational-age (LGA) infants have an increased risk of obesity and autism in life (5–7). Therefore, exploring the effect of these clinical techniques on birth weight is important to ensure the healthy growth of ART offspring. Several studies have shown an increased risk of LGA and high birth weight (HBW) using frozen/thawed embryo transfer compared to fresh transfer (8, 9). Although the effect of an extended embryo culture on birth weight has received much attention in recent years, the results are inconsistent. For fresh cycles, some studies have found significantly higher birth weight and an increase in the number of LGA babies after blastocyst transfer compared with those with cleavage-stage embryo transfer (10, 11), while another two studies did not detect significant differences in birth weight between blastocyst transfer and cleavage-stage embryo transfer in fresh cycles (12, 13). For the effect of culture period on birth weight in frozen embryo transfer cycles, two recent studies with small sample sizes also pointed to the opposite conclusions. The study by Zhang et al. reported that the proportion of LGA infants was higher in singletons born after blastocyst transfer than after transfer of cleavage-stage embryos in vitrified-warmed transfer cycles, but Anick et al. found that the transfer of vitrified blastocysts was associated with a lower birth weight compared with the transfer of vitrified cleavage-stage embryos (14, 15). Although the study performed by Holden et al. explored the different obstetrical and perinatal outcomes using the national database, it included embryos cryopreserved by both the conventional slow freeze technique and vitrification (16). Therefore, with the wide application of vitrification, it is important to evaluate the effect of an extended culture period on birth weight in vitrified-warmed transfer cycles.

Considering the popularity of FET, blastocyst transfer, and SET in clinical applications, and the open question with regard to the effect of an extended culture period on birth weight, we conducted this retrospective cohort study to evaluate the effect of an extended culture period on the birth weight of singletons born after single or double blastocyst transfer compared to cleavage-stage embryo transfer in vitrified-warmed cycles.

## Materials and methods

### Study design and population

This is a retrospective cohort study, conducted in the Shanghai Ninth People’s Hospital affiliated with JiaoTong University School of Medicine (a large hospital-based tertiary care reproductive center in Shanghai, China). Women conceived after the transfer of one or two vitrified-warmed embryos and delivered a singleton baby after at least 20 weeks of gestation from January 2013 to December 2018. Patients were excluded if they had mixed cleavage-stage embryo-blastocyst transfer or if they underwent preimplantation genetic testing (PGT). Women were excluded if more than one gestational sac was detected by transvaginal ultrasound in the first trimester of pregnancy. Furthermore, women were excluded who had missing data on infant sex, birth weight, gestational age, number of transferred embryos and stage of embryo development. For women having more than one singleton birth, only the first birth was included. Additionally, patients with pregnancy-associated syndromes, including pregnancy-related hypertension and diabetes, were excluded, because of their influences on neonatal outcomes. The participant selection procedure is presented in **Supplemetary Figure S1**.

Patients were categorized into four groups: single cleavage-stage embryo transfer (SET-C), single blastocyst transfer (SET-B), double cleavage-stage embryo transfer (DET-C), and double blastocyst transfer (DET-B). This study protocol was approved by the ethics committee of the hospital and was carried out in accordance with the Helsinki Declaration.

### Procedures

Our previous studies have described the procedures in detail for the ovulation induction, IVF/ICSI procedure, and embryo culture, freezing, thawing, and transfer (17–19). IVF or ICSI was performed depending on the semen quality. Normal fertilization was assessed 16-18 hours after insemination/injection. Then the embryos were subsequently cultured and embryo morphology was graded on Day 3 or Day 5/6.

Cleavage-stage embryos were classified as high-quality embryos if they had six to eight blastomeres on Day 3, with fragmentation < 20% according to Cummin’s criteria. Blastocysts scored according to the Gardner and Schoolcraft grading system were recorded as high quality if they reached at least an expansion stage 3 with A or B for inner cell mass and trophectoderm (3BB). After embryo grading, all cleavage-stage embryos and blastocysts were frozen using the vitrification method. In brief, the cryotop carrier system (Kitazato Biopharma Co. Ltd, Japan) was used for vitrification, and 15% (v/v) ethylene glycol, 15% (v/v) dimethylsulfoxide and 0.5 mol/l sucrose were used as the cryoprotectant. For warming, 1.0 mol/l, 0.5 mol/l and 0.0 mol/l sucrose solutions were used for stepwise cryoprotectant dilution. All vitrification and warming steps were carried out at room temperature except for the first warming step, which was at 37 °C. The same continuous single culture media (Irvine Scientific) and vitrification method was employed throughout the whole study period.

Endometrial preparation was performed as previously described (20). A natural cycle was used for patients with regular menstrual cycles, and a hormone therapy cycle or stimulation cycle was used for patients with irregular menstrual cycles. In the natural cycle, we monitored follicular growth by means of serum hormones and transvaginal ultrasound from cycle Day 10 onwards. When the diameter of the dominant follicle was >16 mm and the endometrial thickness was >8 mm, a bolus of hCG was injected to trigger ovulation. In the stimulation cycle, letrozole was prescribed orally for 5 days beginning on cycle Day 3 of menses. In the case of a dominant follicle <14 mm on Day 10, a daily dosage of 75 IU hMG (Anhui Fengyuan Pharmaceutical Co.) was added to stimulate follicle growth and the endometrial lining. In the hormone therapy cycle, oral E2 was commenced on the third day of the menstrual cycle, and progesterone exposure was initiated when the endometrial thickness was appropriate (usually >8 mm). The day of embryo transfer was determined based on the length of the culture period being 3 days or 5/6 days. In all FET cycles, one or two embryos were transferred according to the number of embryos and the patient’s intention under ultrasound guidance, and serum *β*-HCG levels were measured 14 days after embryo transfer.

### Data collection

The data used in the study were from the ART database of our center, which included all records about the basic demographic characteristics of patients, treatment details and outcomes. Details of treatments with ART and any birth resulting from ART have been recorded in the database, which was required by the Technical Standard for Human Assisted Reproduction issued by the Chinese Ministry of Health (CMOH). Variables extracted for this study included the following: maternal age, paternal age, maternal body mass index (BMI), type of infertility (primary infertility or second infertility), parity (nulliparous, pluriparous), duration of infertility, causes of infertility (female factor, male factor, combined factor, and unexplained infertility), fertilization type (IVF-only, ICSI-only, or IVF/ICSI split), sperm origin (ejaculation or testicular sperm extraction), number of transferred embryos (one or two embryos), stage of embryo development (blastocyst or cleavage-stage embryo) and endometrial preparation program (natural cycle, mild stimulation cycle, or hormonal replacement cycle). Information regarding infant sex, birthweight (unadjusted), and gestational age was also obtained.

### Outcomes

Gestational age was calculated by adding 17 days for cleavage-stage embryo transfer and 19 days for blastocyst transfer from the embryo transfer date (14). Outcomes analyzed included very preterm birth (VPTM, <32 weeks of gestation), preterm birth (PTM, <37 weeks of gestation), very low birth weight (VLBW, <1500 g at birth), low birth weight (LBW, <2500 g at birth), high birth weight (HBW, >4000 g at birth), very high birth weight (VHBW, >4500 g at birth), small for gestational age (SGA, defined as birth weight <10^th^ percentile for gestational age using the Chinese reference singleton newborns stratified by gestational age and neonatal sex), very small for gestational age (VSGA, defined as birth weight <3^rd^ percentile), large for gestational age (LGA, defined as birth weight >90^th^ percentile), and very large for gestational age (VLGA, defined as birth weight >97^th^ percentile) (21). The adjusted birth weight (known as gestational age-adjusted and sex-adjusted birth weights) was also calculated, as described in a previous study (14). Birth defects were defined according to the International Classification of Diseases, 10th Revision (ICD-10), and a detailed description can be found in our previously published paper (19, 22).

### Statistical analysis

The baseline characteristics and neonatal outcomes are presented as the mean (standard deviation, SD) for continuous variables and percentage for categorical variables. To investigate the influencing factors on gestational age, unadjusted birth weight, and adjusted birth weight, multivariable linear regression models were performed after controlling for other potential confounders. Logistic regression models were used to compute odds ratios (ORs) and 95% confidence intervals (CIs) for estimating the influencing factors on neonatal outcomes including the same covariates as the linear regression models. All statistical analyses were performed using a two-sided 5% level of significance and the statistical package Stata, Version 12 (StataCorp, USA). P < 0.05 was considered to be statistically significant.

### Ethical approval

This study protocol was approved by the Ethics Committee (Institutional Review Board) of the Shanghai Ninth People’s Hospital (reference number 2017-211).

## Results

A total of 12400 singleton live-born infants were included in this study. Of these, 1015, 1027, 687, and 9671 singletons were obtained after single blastocyst transfer, single cleavage-stage embryo transfer, double blastocyst transfer, and double cleavage-stage embryo transfer, respectively. The maternal and treatment characteristics of singletons are presented in **Table 1**. The average maternal age and paternal age were 31.53 years and 33.45 years, respectively. Slightly more than 50% of women had primary infertility, and the proportion of nulliparous women was above 90%. For the infertility causes, female factors (approximately 60%) were the most common, followed by combined factors. IVF was the main fertilization method.

**Table 1:** Maternal and treatment characteristics of singletons born after single- or double- vitrified cleavage-stage embryo or blastocyst transfer.

Characteristic	SET-B (n=1015)	SET-C (n=1027)	DET-B (n=687)	DET-C (n= 9671)	Total (n=12400)	P_1_	P_2_
Maternal age, mean ± SD	31.76(4.12)	32.14(4.08)	30.68(3.74)	31.50(4.20)	31.53(4.17)	0.039	<0.001
Paternal age, mean ± SD	33.60(5.07)	34.07(5.29)	32.53(4.76)	33.44(5.30)	33.45(5.26)	0.044	<0.001
Maternal BMI, mean ± SD	21.01(4.09)	21.61(3.87)	20.78(3.70)	21.25(4.11)	21.23(4.07)	<0.001	0.004
Type of infertility						0.878	0.341
Primary infertility	548(53.99)	551(53.65)	358(52.11)	5221(53.99)	6678(53.85)		
Second infertility	467(46.01)	476(46.35)	329(47.89)	4450(46.01)	5722(46.15)		
Parity,n(%)						0.449	0.529
Nulliparous	942(92.81)	944(91.92)	638(92.87)	8917(92.20)	11441(92.27)		
Pluriparous	73(7.19)	83(8.08)	49(7.13)	754(7.80)	959(7.73)		
uration of infertility, mean ± SD	3.78(2.64)	3.95(2.77)	3.61(2.32)	3.83(2.66)	3.82(2.65)	0.193	0.046
Infertility causes, n (%)						0.491	<0.001
Female factor	630(62.07)	603(58.71)	466(67.83)	5763(59.59)	7462(60.18)		
Male factor	103(10.15)	112(10.91)	61(8.88)	1191(12.32)	1467(11.83)		
Combined factor	194(19.11)	214(20.84)	104(15.14)	1906(19.71)	2418(19.50)		
Unexplained	88(8.67)	98(9.54)	56(8.15)	811(8.39)	1053(8.49)		
Fertilization type, n (%)						0.552	0.040
IVF-only	635(62.56)	646(62.90)	462(67.25)	6050(62.56)	7782(62.76)		
ICSI-only	272(26.80)	259(25.22)	167(24.31)	2588(26.76)	3286(26.50)		
IVF/ICSI split	108(10.64)	122(11.88)	58(8.44)	1033(10.68)	1332(10.74)		
Sperm origin						0.222	0.214
Ejaculation	994(97.93)	1013(98.64)	676(98.40)	9445(97.66)	11827(97.75)		
Testicular sperm extraction	21(2.07)	14(1.36)	11(1.60)	226(2.34)	272(2.25)		

SET-C, single cleavage-stage embryo transfer; SET-B, single blastocyst transfer; DET-C, double cleavage-stage embryo transfer; DET-B, double blastocyst transfer. BMI, body mass index; IVF, in vitro fertilization; ICSI, intracytoplasmic sperm injection; FET, frozen-thawed embryo transfer.

P<0.05 indicates a statistically significant difference; P_1_: SET-B vs. SET-C, P_2_: DET-B vs. DET-C.

The percentage of birth outcomes among singletons born after single blastocyst transfer, single cleavage-stage embryo transfer, double blastocyst transfer, and double cleavage-stage embryo transfer are presented in **Table 2**. The results of the multivariate analysis of birth outcomes are shown in **Tables 3** and **4** to explore the influencing factors.

**Table 2:** Neonatal outcomes of singletons by number of embryos transferred and stage of embryo development.

	SET-B (n=1015)	SET-C (n=1027)	DET-B (n=687)	DET-C (n=9671)	Total (n=12400)	P1	P2
Newborn gender, n (%)						<0.001	0.274
Female	419(41.28)	514(50.05)	317(46.14)	4671(48.30)	6479(52.25)		
Male	596(58.72)	513(49.95)	370(53.86)	5000(51.70)	5921(47.75)		
Gestational age, mean ± SD	38.83(1.73)	38.77(1.65)	38.90(1.56)	38.94(1.60)	38.92(1.61)	0.471	0.458
Very preterm (<32w), n (%)	10(0.99)	11(1.07)	5(0.73)	89(0.92)	115(0.93)	0.848	0.607
Preterm (<37w), n (%)	89(8.77)	78(7.59)	58(8.44)	662(6.85)	887(7.15)	0.333	0.112
Unadjusted birth weight, mean ± SD	3344.31(533.02)	3308.32(508.35)	3372.04(517.85)	3345.39(500.38)	3343.71(504.85)	0.119	0.178
Adjusted birth weight, mean ± SD	0.30(1.07)	0.23(1.05)	0.35(1.10)	0.28(1.06)	0.28(1.06)	0.142	0.072
Very low birth weight (<1500g), n (%)	11(1.08)	8(0.78)	1(0.15)	55(0.57)	75(0.60)	0.473	0.182
Low birth weight (<2500g), n (%)	47(4.63)	40(3.89)	28(4.08)	403(4.17)	518(4.18)	0.410	0.908
High birth weight (>4000g), n (%)	75(7.39)	53(5.16)	56(8.15)	641(6.63)	825(6.65)	0.038	0.124
Very high birth weight (>4500g), n (%)	7(0.69)	9(0.88)	7(1.02)	83(0.86)	106(0.85)	0.632	0.661
Small for gestational age (<10th percentile), n (%)	60(5.91)	69(6.72)	47(6.84)	584(6.04)	760(6.13)	0.453	0.395
Large for gestation age (>90th percentile), n (%)	178(17.54)	148(14.41)	121(17.61)	1553(16.06)	2000(16.13)	0.054	0.285
Very small for gestational age (<3rd percentile), n (%)	21(2.07)	24(2.34)	16(2.33)	201(2.08)	262(2.11)	0.680	0.658
Very large for gestational age (>97th percentile), n (%)	67(6.60)	54(5.26)	55(8.01)	584(6.04)	760(6.13)	0.199	0.038
Major birth defects, n (%)	8(0.79)	10(0.97)	6(0.87)	65(0.67)	89(0.72)	0.654	0.537

P<0.05 indicates a statistically significant difference; P1: SET-B vs. SET-C, P2: DET-B vs. DET-C.

**Table 3:** Multivariable linear regression analysis for unadjusted birth weight, gestational age, and adjusted birth weight.

	Unadjusted birth weight(g)			Gestational age(weeks)			Adjusted birth weight(g)		
	β	Std. Error	P value	β	Std. Error	P value	β	Std. Error	P value
Blastocyst vs. Cleavage embryo	30.28	13.17	0.022	-0.03	0.05	0.599	0.09	0.03	0.007
Double vs. single embryo	13.93	12.25	0.255	0.11	0.05	0.019	0.05	0.03	0.099
Maternal age	-0.53	1.51	0.727	-0.01	0.01	0.013	0.00	0.00	0.596
Paternal age	0.49	1.16	0.674	-0.01	0.00	0.168	0.00	0.00	0.906
Maternal BMI	10.34	0.97	<0.001	-0.02	0.00	<0.001	0.03	0.00	<0.001
Type of infertility									
Second vs. Primary infertility	44.33	8.87	<0.001	0.00	0.03	0.904	0.09	0.02	<0.001
Parity									
Pluriparous vs. Nulliparous	26.06	16.38	0.112	-0.18	0.06	0.005	0.07	0.04	0.090
Duration of infertility	3.21	2.90	0.269	-0.01	0.01	0.205	0.01	0.01	0.294
Infertility causes									
Female factor(reference)									
Male factor	-11.94	13.52	0.377	0.08	0.05	0.125	-0.03	0.03	0.394
Combined factor	-16.64	10.50	0.113	0.06	0.04	0.145	-0.04	0.03	0.152
Unexplained	-19.92	14.80	0.178	0.15	0.06	0.012	-0.05	0.04	0.156
Fertilization type									
IVF-only (reference)									
ICSI-only	15.00	12.68	0.237	0.00	0.05	0.987	0.04	0.03	0.183
IVF/ICSI split	5.48	20.79	0.792	-0.07	0.08	0.388	0.00	0.05	0.986
Sperm origin									
TESE vs. Ejaculation	-25.05	28.52	0.380	0.15	0.11	0.181	-0.06	0.07	0.453
Endometrial preparation program Natural cycle (reference)									
Mild Stimulation cycle	-12.45	10.01	0.214	-0.16	0.04	<0.001	-0.04	0.03	0.092
Hormonal replacement cycle	14.99	10.48	0.153	-0.19	0.04	<0.001	0.05	0.03	0.069
Neonatal gender									
Female vs. male	-137.93	8.07	<0.001	0.14	0.03	<0.001	-0.07	0.02	0.001
Gestational age	179.04	2.53	<0.001	—	—	—	0.10	0.01	<0.001

As presented in **Table 2**, the mean gestational age was 38.83 weeks for single blastocyst transfer, 38.77 weeks for single cleavage-stage embryo transfer, 38.90 weeks for double blastocyst transfer, and 38.94 weeks for double cleavage-stage embryo transfer. The developmental stage of transferred embryos was not significantly associated with gestational age by multivariable linear regression analysis, as shown in **Table 3**. The mean unadjusted birth weights were 3344.31 g, 3308.32 g, 3372.04 g, and 3345.39 g for singletons born after single blastocyst transfer, single cleavage-stage embryo transfer, double blastocyst transfer, and double cleavage-stage embryo transfer, respectively, as shown in **Table 2**. After adjusting for several important variables, the multivariable linear regression results in **Table 3** showed that the unadjusted birth weights of singletons born after vitrified blastocyst transfer were higher than those after cleavage-stage transfer (β=30.28, SE=13.17, P=0.022), as were the adjusted birth weights (β=0.09, SE=0.03, P=0.007). In addition, birth weight was positively related to maternal BMI, and male newborns had higher birth weights than female newborns, as showed in **Table 3**.

Furthermore, **Table 2** shows that the proportions of infants with high birth weight were 7.39%, 5.16%, 8.15%, and 6.63% among singletons born after single blastocyst transfer, single cleavage-stage embryo transfer, double blastocyst transfer, and double cleavage-stage embryo transfer, respectively, and the corresponding proportions for infants with LGA were 17.54%, 14.41%, 17.61%, and 16.06%. After adjusting for the known confounding factors by logistic regression analysis, as shown in **Table 4**, there was a 37% increased odd of having an infant with high birth weight after vitrified blastocyst transfer compared with vitrified cleavage stage transfer (OR=1.37, 95% CI:1.07-1.77). However, the number of transferred embryos was not observed to be related to HBW or LGA.

**Table 4:** Logistic regression analysis for neonatal outcome according to number and stage of embryo transfer among singletons.

	LBW OR (95%CI)	HBW OR (95%CI)	SGA OR (95%CI)	LGA OR (95%CI)
Blastocyst vs. Cleavage embryo	1.01(0.65,1.56)	1.37(1.07,1.77)	0.96(0.73,1.26)	1.15(0.97,1.37)
Double vs. single embryo	1.34(0.89,2.06)	1.22(0.94,1.56)	1.00(0.78,1.29)	1.06(0.90,1.24)
Maternal age	1.01(0.96,1.06)	0.97(0.94,1.00)	1.01(0.98,1.04)	0.98(0.96,1.00)
Paternal age	1.01(0.97,1.05)	1.02(0.99,1.04)	0.99(0.96,1.01)	1.01(0.99,1.02)
Maternal BMI	0.98(0.95,1.01)	1.12(1.10,1.15)	0.98(0.96,1.00)	1.08(1.05,1.09)
Type of infertility				
Second vs. Primary infertility	0.99(0.73,1.34)	1.16(0.98,1.39)	0.88(0.73,1.05)	1.21(1.07,1.36)
Parity				
Pluriparous vs. Nulliparous	0.79(0.46,1.35)	1.33(0.98,1.79)	0.81(0.56,1.17)	1.17(0.95,1.43)
Duration of infertility	1.03(0.93,1.13)	0.98(0.93,1.04)	1.00(0.95,1.06)	1.00(0.96,1.04)
Infertility causes				
Female factor(reference)				
Male factor	1.35(0.85,2.14)	0.83(0.62,1.10)	1.04(0.79,1.37)	1.00(0.83,1.19)
Combined factor	1.24(0.88,1.75)	0.89(0.72,1.10)	1.15(0.93,1.41)	0.95(0.83,1.09)
Unexplained	1.31(0.79,2.18)	0.89(0.66,1.20)	1.13(0.84,1.52)	0.95(0.78,1.16)
Fertilization type				
IVF-only (reference)				
ICSI-only	1.09(0.70,1.68)	0.96(0.75,1.24)	0.99(0.77,1.29)	1.00(0.84,1.18)
IVF/ICSI split	0.61(0.30,1.24)	1.25(0.83,1.89)	0.97(0.64,1.48)	1.10(0.84,1.45)
Sperm origin				
TESE vs. Ejaculation	0.11(0.02,0.60)	1.21(0.69,2.11)	0.70(0.36,1.37)	0.81(0.54,1.22)
Endometrial preparation program Natural cycle (reference)				
Mild Stimulation cycle	1.10(0.77,1.58)	0.83(0.68,1.02)	0.97(0.79,1.19)	0.92(0.80,1.05)
Hormonal replacement cycle	1.10(0.76,1.58)	1.10(0.90,1.34)	0.93(0.75,1.16)	1.13(0.99,1.30)
Neonatal gender				
Female vs. male	1.54(1.16,2.03)	0.56(0.48,0.66)	1.26(1.07,1.49)	0.96(0.87,1.07)
Gestational age	0.31(0.29,0.34)	1.69(1.56,1.83)	0.84(0.80,0.87)	1.14(1.10,1.19)

OR, odds ratio; CI, confidence interval.

## Discussion

To the best of our knowledge, this is the largest study to explore the effect of extended embryo culture on the birth weight of singletons born after single or double vitrified-warmed embryo transfer. This study showed a significant increase in the unadjusted and adjusted birth weight among singletons born after vitrified blastocyst transfer compared with singletons born after vitrified cleavage-stage embryo transfer after controlling for confounders. Similarly, the results suggested an increased odds of having an infant with HBW after vitrified blastocyst transfer. In addition, only pregnancies with a single gestational sac after single or double embryo transfer were included in the study and no significant differences were found in neonatal outcomes between singletons born after single embryo transfer and singletons born after double embryo transfer.

With advances in embryo culture conditions, extending embryo culture to the blastocyst stage is increasingly used clinically as an effective operation method to reduce the multiple pregnancy rate and improve the implantation rate and live birth rate. Considering that ultimate outcome of ART is a healthy singleton baby, the neonatal outcome was more appropriate as a measure to evaluate effectiveness and safety. In recent years, a few studies on the influences of an extended embryo culture on neonatal outcomes have been conducted. However, many existing studies were performed in fresh embryo transfer cycles (10–13). Even though a few studies focused on frozen embryo transfer, their findings were conflicting and limited by the small sample size or the inclusion of both the slow freeze technique and vitrification method (14–16). Therefore, we conducted this larger study focusing on vitrified-warmed cycles to evaluate the neonatal outcomes after blastocyst transfer. In the present study, the unadjusted and adjusted birth weights for singletons born after vitrified blastocyst transfer were significantly heavier than those born after cleavage-stage embryo transfer, and the risk of delivery of an HBW infant after vitrified blastocyst transfer also increased. This result indicated that blastocyst transfer had a significant impact on birth weight, which was in agreement with some previous studies (11, 14). Considerable evidence in animal models also showed that the exposure of ovine and bovine embryos to *in vitro* culture conditions prior to the blastocyst stage could induce large offspring syndrome (23). The epigenetic reprogramming of the embryo genome in the culture period may be a possible mechanism to explain the significant increase in birth weight after blastocyst transfer. Published studies have reported that epigenetic mechanisms affect neonatal weight (24, 25). Exposure to an *in vitro* culture environment as a perturbation for preimplantation embryo development altered the expression of imprinted genes regulating fetal growth and development by changing DNA methylation (26). Li et al. identified 320 genomic loci alterations in DNA methylation in large offspring syndrome produced by assisted reproduction in bovines. Importantly, their study showed that large offspring syndrome in bovines shares phenotypes and epigenotype aberrations with the most common congenital overgrowth syndrome in humans. Therefore, alterations in DNA methylation during embryo culture are potential mechanisms for explaining the phenomenon in this study (27).

In our study, after controlling for other variables, the birth weight, and odds of HBW or LGA infants were not different between singletons born after single embryo transfer and singletons born after double embryo transfer. The above results indicated that the number of transferred embryos was not related to neonatal outcomes among singletons. However, the different birth weights among singletons after single embryo transfer and double embryo transfer have been reported in a previous study (28). Sutter reported that singletons born after double embryo transfer had a significantly lower birth weight than that of singletons born after single embryo transfer (28). Early vanishing twins, as the important factor, could induce a lower birth weight, which may explain the contradictory results between the study by Sutter and this study.

Many factors can affect birth weight. In the multivariate regression analysis, many variables, such as maternal age, paternal age, maternal BMI, type of infertility, parity, duration of infertility, infertility causes, fertilization type, sperm origin, gestational age and neonatal sex, were included to explore their relationships with birth weight. Consistent with previous reports, birth weight significantly increased with maternal BMI and gestational age, and the birth weight of male infants was significantly heavier than that of female infants.

The main strength of the present study was that it had the largest sample size to date, which enhanced the findings in the study. The second strength was that the same embryo culture medium was used during the study period, which avoids the adverse influence of different culture media on neonatal birth weight (29). In addition, we included only vitrified-warmed embryo transfer cycles to avoid the adverse effects of the hyperestrogenic milieu on neonatal outcomes. However, there were several limitations in our study. First, a higher birth weight among singletons born after transfer of vitrified-warmed blastocysts compared to vitrified-warmed cleavage stage embryos was demonstrated. However, the size was rather small compared with the higher birth weight of frozen versus fresh blastocyst transfer (9). Further studies are warranted to clarify the cause of the differences. Second, slight difference exists in the vitrification process between cleavage-stage embryos and blastocysts. Cleavage-stage embryos can be frozen directly, while blastocysts need to be manually shrunk prior to vitrification. It remains unclear whether this difference will yield potential consequences for the birth weight of future offspring (30, 31). Therefore, we cannot rule out the possibility that the higher birth weight is due to a greater effect of the vitrification-thawing process itself on the blastocyst compared to the cleavage-stage embryo, and a randomized study of Day 3 versus Day 5/6 using fresh embryo transfer would be necessary.

## Conclusion

In conclusion, this study showed that unadjusted and adjusted birth weights significantly increased after blastocyst transfer compared with cleavage-stage embryo transfer in vitrified-warmed cycles. In addition, blastocyst transfer was significantly associated with an increased odds of having an infant with HBW. Considering the increasing trend towards blastocyst transfer and the freeze-all strategy and a healthy singleton birth as the ultimate goal of IVF, this study provided evidence about the potential clinical safety issues for offspring from blastocyst transfer in vitrified-warmed embryo cycles.

## Data availability statement

The original contributions presented in the study are included in the article/**Supplementary Material**. Further inquiries can be directed to the corresponding authors.

## Ethics statement

The studies involving human participants were reviewed and approved by the Ethics Committee (Institutional Review Board) of the Shanghai Ninth People’s Hospital. Written informed consent for participation was not required for this study in accordance with the national legislation and the institutional requirements.

## Author contributions

NW drafted the manuscript. KL and XZ collected patient data. XZ and PZ conceived and designed the study. All authors contributed to the article and approved the submitted version.
