# Supplementary figures and images for: The effect of an extended culture period on birth weight among singletons born after single or double vitrified embryo transfer
Source: Front Endocrinol (Lausanne). 2024 Mar 19;15:1184966. doi: 10.3389/fendo.2024.1184966 (PMC10985195; doi:10.3389/fendo.2024.1184966)

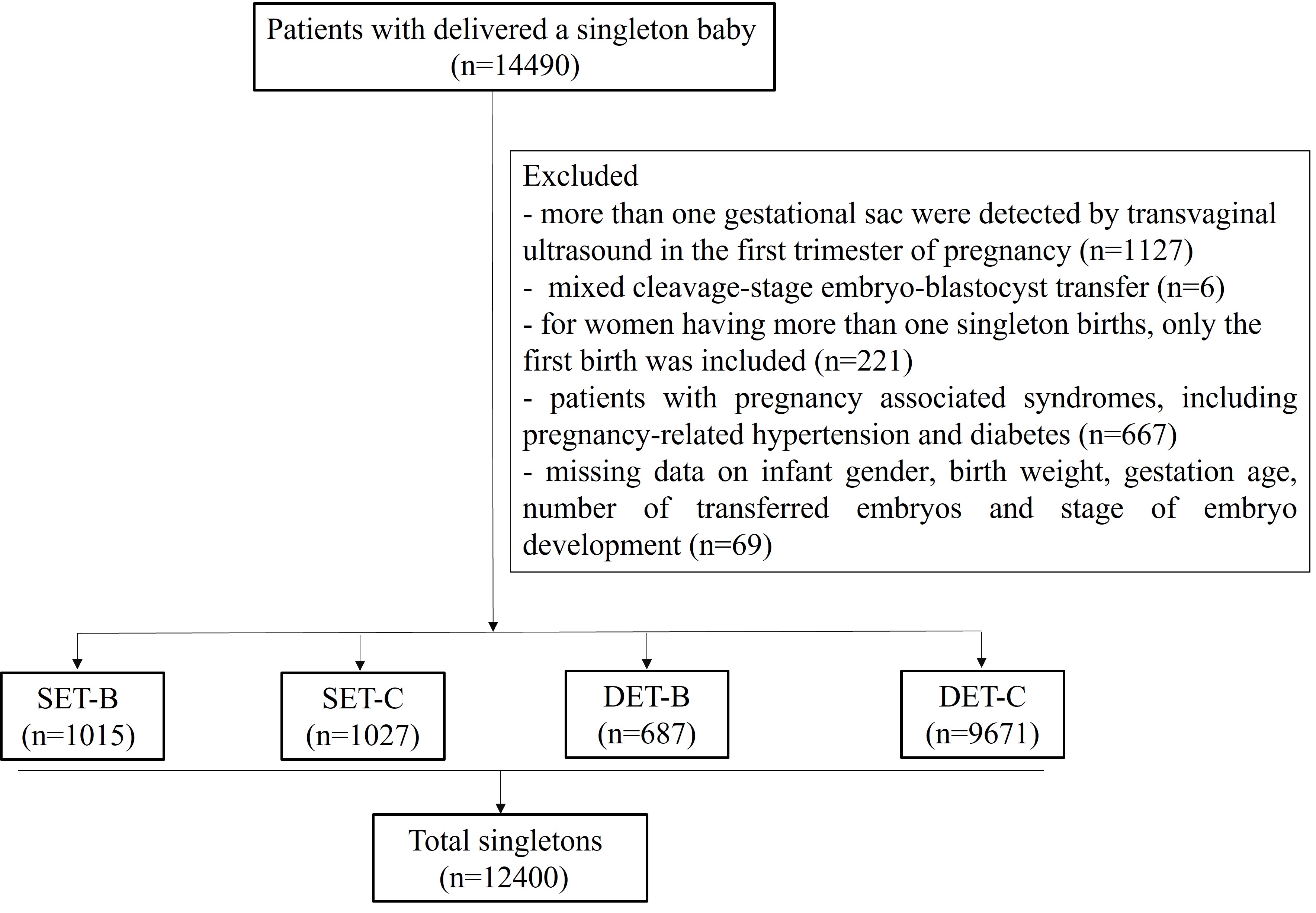

Supplement: Supplementary file 1 [file Image_1.jpeg]
